# Overview of methodologies for T-cell receptor repertoire analysis

**DOI:** 10.1186/s12896-017-0379-9

**Published:** 2017-07-10

**Authors:** Elisa Rosati, C Marie Dowds, Evaggelia Liaskou, Eva Kristine Klemsdal Henriksen, Tom H Karlsen, Andre Franke

**Affiliations:** 1 Institute of Clinical Molecular Biology, Kiel University, Rosalind-Franklin-Str. 12, 24105 Kiel, Germany; 2 Centre for Liver Research and NIHR Birmingham Liver Biomedical Research Unit, Institute of Immunology and Immunotherapy, University of Birmingham, Birmingham, UK; 3 Norwegian PSC Research Center, Department of Transplantation Medicine, Division of Surgery, Inflammatory Medicine and Transplantation, Oslo University Hospital Rikshospitalet, Oslo, Norway; 4 Research Institute of Internal Medicine, Division of Surgery, Inflammatory Medicine and Transplantation, Oslo University Hospital Rikshospitalet, Oslo, Norway; 5 K.G. Jebsen Inflammation Research Centre, Institute of Clinical Medicine, University of Oslo, Oslo, Norway; 6 Institute of Clinical Medicine, Faculty of Medicine, University of Oslo, Oslo, Norway; 7 Section of Gastroenterology, Department of Transplantation Medicine, Division of Surgery, Inflammatory Medicine and Transplantation, Oslo University Hospital Rikshospitalet, Oslo, Norway

**Keywords:** T-cell receptor (TCR), TCR profiling, TCR repertoire, Immune repertoire, Immunogenetics, Immunogenomics, Vdj, CDR3, Clonotype, Target sequencing

## Abstract

**Background:**

The T-cell receptor (TCR), located on the surface of T cells, is responsible for the recognition of the antigen-major histocompatibility complex, leading to the initiation of an inflammatory response. Analysing the TCR repertoire may help to gain a better understanding of the immune system features and of the aetiology and progression of diseases, in particular those with unknown antigenic triggers. The extreme diversity of the TCR repertoire represents a major analytical challenge; this has led to the development of specialized methods which aim to characterize the TCR repertoire in-depth. Currently, next generation sequencing based technologies are most widely employed for the high-throughput analysis of the immune cell repertoire.

**Results:**

Here, we report on the latest methodological advancements in the field by describing and comparing the available tools; from the choice of the starting material and library preparation method, to the sequencing technologies and data analysis.

Finally, we provide a practical example and our own experience by reporting some exemplary results from a small internal benchmark study, where current approaches from the literature and the market are employed and compared.

**Conclusions:**

Several valid methods for clonotype identification and TCR repertoire analysis exist, however, a gold standard method for the field has not yet been identified. Depending on the purpose of the scientific study, some approaches may be more suitable than others. Finally, due to possible method specific biases, scientists must be careful when comparing results obtained using different methods.

**Electronic supplementary material:**

The online version of this article (doi:10.1186/s12896-017-0379-9) contains supplementary material, which is available to authorized users.

## Background

**Fig. 1:**
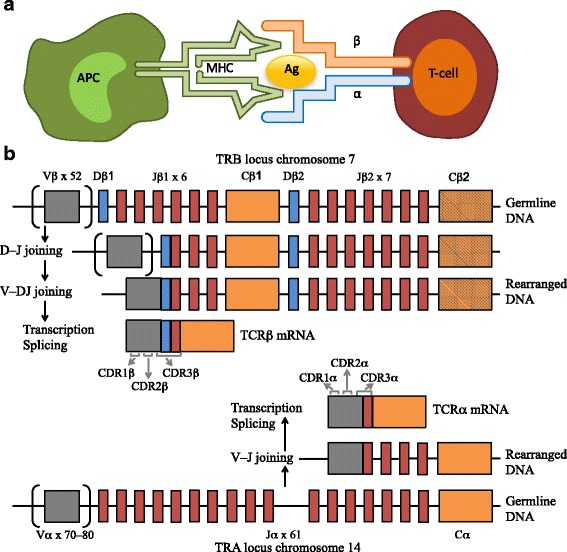
Interaction between an antigen presenting cell (APC) and a T cell, and V(D)J recombination. **a** Interaction between the antigen–major histocompatibility complex (MHC) and the αβ T-cell receptor (TCR). **b** V(D)J recombination: During T cell development, the loci that encode T-cell receptor α and β-chains are rearranged. For both loci, variable (V) and joining (J) gene segments, and an additional diversity (D) gene segment for the β-chain, are recombined to form the final rearranged TCR DNA sequence. This process also involves the deletion and insertion of nucleotides at the V-D, D-J and V-J junctions (not shown). Following transcription, the sequence between the recombined V (D)J regions and the gene encoding the constant (C) region is removed by splicing. The complementarity-determining region (CDR) 3 is encoded by the V (D) J junction, whereas the CDR1 and CDR2 loops are encoded within the germline V gene

T cell mediated antigen recognition depends on the interaction of the T-cell receptor (TCR) with the antigen-major histocompatibility complex (MHC) molecules (Fig. 1a). TCRs are highly diverse heterodimers, consisting of a combination of α and β chains (αβ TCR) expressed by the majority of T cells, or γδ chains (γδ TCR) expressed by T cells in peripheral blood (1–5%) and T cells found at mucosal sites [1]. Similar to immunoglobulins expressed by B cells – membrane bound immunoglobulins are often referred to as B-cell receptors (BCRs) – the TCR chains consist of a variable region, important for antigen recognition, and a constant region. The variable region of TCRα and δ chains is encoded by a number of variable (V) and joining (J) genes, while TCRβ and γ chains are additionally encoded by diversity (D) genes [2, 3]. During VDJ recombination, one random allele of each gene segment is recombined with the others to form a functional variable region (Fig. 1b). Recombination of the variable region with a constant gene segment results in a functional TCR chain transcript. Additionally, random nucleotides are added and/or deleted at the junction sites between the gene segments. This process leads to strong combinatorial (depending on which gene regions will recombine) and junctional diversity (which and how many nucleotides will be added/deleted), resulting in a large and highly variable TCR repertoire, which will ensure the identification of a plethora of antigens. Additional diversity is achieved by the pairing of α and β or γ and δ chains to form a functional TCR [4].

Each TCR chain contains three hypervariable loops in its structure, termed complementarity determining regions (CDR1–3). CDR1 and 2 are encoded by V genes and are required for interaction of the TCR with the MHC complex. CDR3, however, is encoded by the junctional region between the V and J or D and J genes and is therefore highly variable. It plays an essential role in the interaction of the TCR with the peptide-MHC complex, as it is the region of the TCR in direct contact with the peptide antigen. For this reason, CDR3 is often used as the region of interest to determine T cell clonotypes, as it is highly unlikely that two T cells will express the same CDR3 nucleotide sequence, unless they have derived from the same clonally expanded T cell [2, 4].

The sum of all TCRs by the T cells of one individual is termed the TCR repertoire or TCR profile. The TCR repertoire can change greatly with the onset and progression of diseases, which is why scientists are becoming more and more interested in determining the immune repertoire status under different disease conditions, such as cancer, autoimmune, inflammatory and infectious diseases. For example, Muraro et al. used the TCR repertoire to analyse the effect of autologous stem cell transplantation on T cell populations in multiple sclerosis patients [5]. In cancer, cytotoxic T cells can kill tumour cells upon recognition of tumour specific antigens. Some studies have tried to identify specific T cell clonotypes involved in this process by analysing tumour infiltrating lymphocyte repertoires [6–8].

The main challenge while studying the immune repertoire is its diversity. VDJ recombination of the different TCR genes could theoretically generate between 10^15^ and 10^20^ TCR chains. Despite this, the actual diversity present in a human body is estimated at around 10^13^ different clonotypes [9], implying that the afore-described seemingly random TCR development is obviously not random at all and is subject to different constraints. Moreover, while there are TCRs that are common in the general population, recent high-resolution studies have shown that the majority of TCRs is rare (in analogy to common vs. rare genomic variants) [10, 11]. This is one of the reasons why precise methods are necessary to properly investigate complete individual immune-repertoires. In the past decades, different techniques were developed which enabled the study of the immune repertoire. Monoclonal antibodies allowed the analysis of specific V gene subgroups by fluorescence microscopy or flow cytometry, while quantitative polymerase chain reaction (PCR) strategies, in parallel with spectratyping techniques, were able to provide a rather low-resolution overview of the repertoire [12]. Despite these methods, for many years technical limitations made it difficult to create a comprehensive overview of real human TCR repertoires, until highly specific methods based on next generation sequencing (NGS) were developed, facilitating the parallel analysis of millions of TCR sequences. Nevertheless, it is still difficult to define a gold standard method, as every available method has its advantages and disadvantages.

In this article, we provide an overview of the currently available methodologies for TCR repertoire analysis, and we also describe the different aspects that a scientist should consider when choosing the appropriate method for the research question to be answered. We performed a small benchmark experiment that comprises some but not all available protocols for NGS-based immune repertoire analysis, and while our benchmark is by no means comprehensive and exhaustive, our results highlight some characteristics of the different methodologies and the approach in general that may serve as a guide for scientists that are interested and new in the field of immunogenetics.

Some publications focusing on immune repertoire profiling are already available and constitute an important source of information for any scientist interested in this research area [12–19]. However, direct comparisons and benchmarks of the most common methods are scarce.

### Choosing the right starting material for TCR profiling

One of the most basic, yet important decisions a scientist should make when choosing a method for TCR analysis regards the starting material, i.e. whether to use genomic DNA (gDNA) or RNA. As discussed previously [14, 20], either starting material has advantages and disadvantages. The points in favour of gDNA are the higher stability and the presence of a single template per cell, which allows for better quantification of single TCR clones [21]. However, using gDNA does not provide any information on the expression level of the genes of interest and may lead to errors in the sequencing results due to introns, possible residuals of VDJ rearrangements and interfering priming sites found in the sample. When using RNA, quantification of single TCR clones is more challenging as a cell will contain multiple TCR transcripts. Many current methods, however, are designed for RNA as starting material, as the studied mRNA contains the final TCR products. Employing RNA potentially allows for sequencing of the entire J and V gene and it provides information about expression levels. Also, the quantity of starting material is a factor that needs to be taken into account. If only low quantities of starting material are available, this can be a limiting factor when selecting a suitable method, as some kits require a minimum input quantity and concentration of RNA or gDNA.

In general, due to the complexity of the target and the threat of batch effects that can affect the downstream data analysis, it is essential to ensure that the processing of all samples is as uniform as possible, for example by using the same concentration of starting material and trying to have a comparable number of reads for each sample [22].

### TCR sequencing

In this article, we focused on the latest high-throughput sequencing (HTS) methods currently available for TCR repertoire profiling. The two options that we discuss are bulk sequencing of pooled immune cell populations or approaches allowing the analysis at the single cell level. We chose to concentrate on protocols for Illumina sequencing platforms, as this is the most widely established technology. Nonetheless, methods compatible with IonTorrent [23–25] and Roche 454 [26–28] exist.

While both methods for the analysis of single cells and cell populations (“bulk methods”) are available, we mainly focused on the latter, which are more commonly used to study TCR diversity and compare distinct repertoires in larger cohorts. The main disadvantage of bulk sequencing is that it can only provide information about the frequency of single TCR chains, but not their pairing. Single cell approaches are therefore becoming more and more important in immune repertoire studies, as they can accurately identify the pairs of the two TCR chains (αβ, γδ) at the cellular level, bringing repertoire analysis to a higher level of complexity. It is also the concrete chain pair that more accurately reflects the biological in vivo function. However, single cell sequencing approaches are currently more expensive, they cover often only a limited number of cells, as compared to bulk approaches, and, they require fresh material for the isolation and sorting of live cells, which is not always available especially when dealing with human diseased material. Some of the aspects we will consider in following paragraphs include the processing of RNA or gDNA samples prior to sequencing (library preparation) and choosing the adequate sequencing depth – the number of replicate reads necessary to efficiently detect the sequences of interest.

### Bulk methods

There are different aspects to consider when choosing population-based TCR analysis methods. The methods we are going to compare differ in many aspects, from the type of starting material (gDNA or RNA) for the library preparation approach and the sequencing method. Some commercial companies offering immune repertoire analyses services are listed in Table 1. Our comparison mainly considers different library preparation methods, for which we will also discuss the different potential biases*.*

**Table 1:** Exemplary companies providing immune repertoire products and services

Company	Service/Kit	Starting material	Library preparation approach	Chains	CDR regions	Organism	Sequencing platform and length (bp)
BGI (Shenzhen, China)	Service	gDNA RNA	Mplex-PCR: primers V- C genes	TCRαTCRβ BCRH, BCRL	CDR3	Human	Illumina: HiSeq2000/2500 (100 × 2 bp) MiSeq (150/300 × 2 bp) Roche454
		RNA	5’RACE	TCRα, TCRβ	CDR1 CDR2 CDR3		
Adaptive Biotechnologies-ImmunoSeq (Seattle, USA)	Service Kit	gDNA cDNA	Mplex-PCR: primers V-J genes	TCRα, TCRβ, TCRδ, TCRγ, BCRH, BCRL, BCRK	CDR3	Human Mouse	Illumina: HiSeq, MiSeq
iRepertoire, Inc. (Huntsville, USA)	Service Kit	gDNA	Mplex-PCR: primers V-J genes	TCRβ	CDR3	Human	Illumina: HiSeq, MiSeq (100/150 × 2 bp)
		RNA	Mplex-PCR: primers V-C genes	TCRα, TCRβ, TCRδ, TCRγ, BCRH, BCRL	CDR2 CDR3	Human Mouse	Illumina: HiSeq, MiSeq (100/150/250 × 2 bp) Roche454 (500 bp)
Clonotech Takara Bio USA, Inc. (Mountain View, USA)	Kit	RNA	SMART technology (5’RACE)	TCRα, TCRβ	CDR1 CDR2 CDR3	Human Mouse	Illumina: HiSeq, MiSeq Used by company for validation: MiSeq (300 × 2 bp)

Many different features are available; these can be combined in more than one way. Choice of primers, sequencing platform and depth may vary depending on starting material and desired outcome. Adaptive Biotechnologies only uses cDNA for limited applications. The company applies primer concentration controls for amplification bias correction and different options for sequencing depth are available (survey, deep, ultra-deep, max depth). iRepertoire may offer sequencing of the CDR2 region, depending on the chosen sequencing length

### Choosing target sequences: Chains and CDR regions

Several companies offer library preparation and sequencing services for all TCR chains, but α chain and β chain remain the most common targets, as αβ T cells constitute the majority of the total T cell population [29]. Historically, the β chain was the main target studied due to its higher combinatorial potential compared to the α chain, which is due to the presence of the D gene component [14]. The β chain is also unique in each single cell, whereas it is possible that two α chains are expressed by the same cell, increasing the level of complexity [30]. γδ T-cell receptors are not widely studied, as γδ T cells only account for a small proportion of the total T cell population. The overall diversity of γδ TCRs is lower, compared to αβ TCRs, and there is an abundance bias based on which anatomical location is being analysed, as γδ T cells are found at higher frequency at mucosal sites. Therefore, they have been of less interest as peripheral blood samples are most widely studied [1]. PCR-based methods may amplify α and β chains simultaneously, but they are often separated and treated as two different samples in the last steps of the library preparation and during sequencing. This has been found to increase the precision and specificity of the outcome [31–33].

The CDR3 region is the preferential target of many TCR repertoire studies, due to its relevance for TCR-peptide interaction. To date, CDR1 and CDR2 have not attracted the same attention from the scientific community, because they do not directly interact with the antigen. However, CDR1 and CDR2 play an important role in making contact with the MHC molecule and thus influence the sensitivity and affinity of the TCR binding [34, 35]. Being aware of the sequence of the entire transcript, including CDR1 and CDR2, may be a great advantage for modelling the TCR structure and its binding properties. Not all methods are able to detect CDR1 and CDR2. This limitation applies especially to protocols using multiple primer sequences. Indeed, many allele-specific primers are designed in different positions of the V genes, often eliminating the possibility of sequencing outside CDR3.

### Library preparation approach

**Fig. 2:**
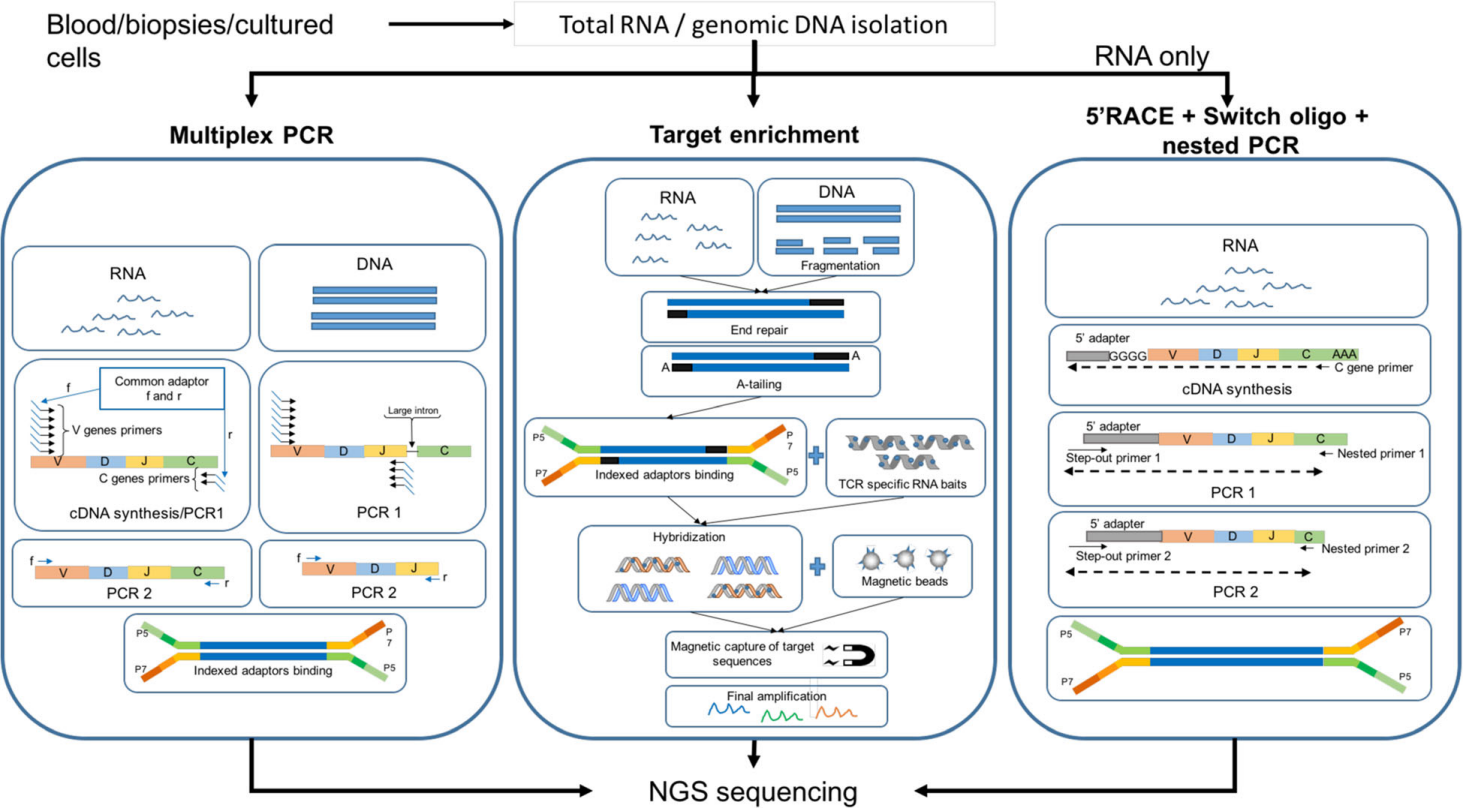
Exemplary workflow of three principal methodologies for TCR library preparation. The figure depicts a simplified workflow of the library preparation procedure using multiplex PCR, targeted in-solution enrichment and 5’RACE-switch-oligo nested PCR. Multiplex PCR is suitable for both RNA and gDNA sequencing. Samples undergo cDNA synthesis and 1 or more PCR steps followed by adaptor ligation and sequencing. While the forward primers for cDNA synthesis are designed to cover all known V genes for both starting materials, the location and number of the reverse primers differs, due to introns in DNA. Target enrichment, also applicable to both gDNA and RNA, is preceded by a standard library preparation including fragmentation for gDNA or mRNA purification for RNA, followed by end-repairing, A-tailing and finally adaptor ligation. The enrichment of target sequences is then performed using RNA baits complementary to the sequence of interest. The RNA baits hybridize with molecules in the library, which are then retrieved using magnetic beads and can undergo further amplification before sequencing. Nested PCR based on the 5’RACE and switch-oligo approach (only for RNA) makes use of the incorporation of an adaptor molecule at the 5′ end of the cDNA during cDNA synthesis. The forward primer for a subsequent PCR is designed to bind to the 5′ adaptor sequence, while the reverse primer is designed to bind to the C-region of the transcript. Hence, only one primer pair is required to cover the complete spectrum of possible V genes. Subsequent nested PCRs performed in the same fashion may increase outcome specificity. Finally, adaptor ligation is performed. The procedures showed in this picture constitute only an example of the different available methods

We regard the library preparation approach as one of the key features to be considered when selecting a method. There are only a few techniques widely used for bulk analysis (Fig. 2). Most published methods are a variant of one of these approaches, the majority of which are PCR-based. Immune repertoire extrapolation from data generated through transcriptome sequencing is also feasible [36]; even though to date it has not been widely used and, given common transcriptome sequencing depths, this approach may be limited and reveal only a fraction of the TCR diversity as compared to a target-specific method.

### Multiplex PCR

Given the diversity of the target, multiplex PCR approaches are among the most widespread, also offered as customizable service or kits by companies such as Adaptive Biotechnologies, BGI and iRepertoire [5, 31, 32, 37–41]. Primers for the J alleles or the constant region of the TCR α and β chains are used together with a mix of primers for all known V alleles. This results in a specific amplification of the TCR transcript across the CDR3 region. The multiplex approach can be used for both gDNA and RNA and the published protocols assure no cross-primer interference during amplification. However, this method cannot detect new V alleles variants due to the fixed set of primers used. Furthermore, multiplex PCR methods are subjected to amplification biases [42], which lead to better amplification of some alleles compared to others, thereby distorting the relative abundances of the resulting products. It is possible to correct for this kind of error by using a specific experimental design including adjustment of primer concentrations [43] and/or using molecular barcoding [44].

### Target enrichment

A targeted enrichment method is available using e.g. Agilent’ RNA baits for capturing TCRs of αβ T cells. For starting material, gDNA or RNA is first processed with a standard sequencing library preparation kit (i.e. Illumina TruSeq or SureSelectXT from Agilent), followed by incubation of the samples with custom designed RNA baits. These RNA baits, which are complementary to the sequences of interest and tolerate a few different bases compared to the target, hybridize with the gDNA/cDNA target, allowing then for capturing it, and submitting the captured gDNA/cDNA to a further amplification step of the wanted sequences. This method, among other approaches, requires fewer PCR cycles and is thus less susceptible to PCR bias. Also α and β chains can be processed together, while it is suggested to separate the processing of the two chains for other methods in order to increase the quality of the outcome [45, 46].

### 5’RACE cDNA synthesis and nested PCR

For RNA samples, rapid amplification of 5′ complementary DNA ends (5’RACE) [47] employing the template-switch effect is becoming a gold standard for bulk TCR analysis [28, 48–50]. This method, marketed by Clonotech as “SMART” technology, relies on the terminal transferase activity of the reverse transcriptase enzyme, which incorporates additional nucleotides (usually dCTP) at the 3′ end of the cDNA molecule during the first strand synthesis reaction. A template-switch oligonucleotide containing an oligo(rG) sequence anchors to the non-template stretch of the first-strand cDNA, allowing the reverse transcriptase to switch templates and to continue replicating to the end of the oligonucleotide [51]. This enables the synthesis of cDNA strands containing the complete 5′ end of the mRNA, independent of the carried V allele, which enables capturing of all TCR variants present in the sample, provided that the integrity of the transcripts is conserved. Recently, Clonotech developed a commercial kit for TCR analysis using the afore-described template-switch technology. cDNA synthesis is carried out using primers against a small proportion of the target TCR mRNA transcript, the constant region. Consecutive PCRs may be carried out using a common adaptor as 5′ primer and constant region primers for the 3′ end. Ideally, these primers should be designed on nested sequences of the constant region in order to increase amplification specificity. Hence, only one primer set is required per reaction, avoiding the use of multiple primer sets and thus the associated amplification bias. The PCR products can be ligated to the appropriate sequencing adaptors and used for NGS sequencing.

### General issues of TCR analysis

Despite the successful adoption and improvements of the mentioned approaches, every method based on PCR is still susceptible to a number of errors intrinsic to the particular technique, namely variable amplification efficiency due to differences in GC content, amplification stochasticity, template-switching and polymerase errors [52]. In addition, sequencing errors independent of the library preparation method used must always be taken in account. TCR sequencing is particularly vulnerable to sequencing errors, since a specific TCR may differ from another by only a single nucleotide. This fact makes it important to distinguish between PCR errors, sequencing errors and low frequency clonotypes. Thus, different techniques have been recently developed to overcome this issue. These methods comprise the usage of unique molecular identifiers (UMIs) introduced during cDNA synthesis to distinguish between single RNA molecules and minimize the impact of PCR amplification and sequencing errors [53], and specific algorithms for correction of this particular type of data [54].

### Unique molecular identifiers

UMIs deserve special attention. The introduction of UMIs enabled the determination of the absolute count of RNA transcripts processed in a sample. UMI sequences, containing random nucleotides, are inserted into the template-switch oligonucleotide, which ligates with target molecules during cDNA synthesis, thereby uniquely barcoding every cDNA molecule in the sample with a different UMI. During data analysis, this allows retrieval of sequences originated from the same mRNA molecule after PCRs and sequencing. In a field like immune repertoire analysis, where target molecules may differ only by a single base, making the distinction between technical errors and biological differences is even more important; thus, using UMIs allows for a straightforward error correction. For these reasons their usage is increasing in immune repertoire profiling practice and herewith recommended [53, 55].

### Sequencing platform and sequencing depth

Some sequencing platforms are more error prone than others, which means that a careful choice of the sequencing depth is needed in order to effectively manage the error rate, especially when not using molecular identifiers. A high sequencing depth allows for analysis of a more complete and complex repertoire [15], but deep sequencing is not always the best choice in immune repertoire analysis, depending on the purpose of the study. Disease-oriented analyses often look for highly expressed and clonally expanded TCRs. In this case, a superficial low-coverage screening of the immune repertoire may even be enough to catch the most common and expanded clonotypes in the sample. For this purpose, the Illumina MiSeq platform is commonly used, while the Illumina HiSeq is more often used for deep sequencing [14]. A general recommendation in order to achieve sufficient coverage for all sequences is to aim for at least 30,000 on-target reads, but ideally 100,000 reads per 10 ng of total starting RNA material (which relates to approximately 10,000 lymphocytes) should be performed [48].

Another issue that should be considered when selecting a sequencing platform is the diversity present in the library. Multiplex PCR and target enrichment based libraries have a higher diversity compared to 5’RACE-based libraries, which may all start with the same adaptor sequence at the 5′ end. Higher diversity makes sequencing with Illumina platforms easier. In case of low diversity samples, as 5′ RACE, some adjustments are necessary to improve the sequencing outcome. Including higher percentages of PhiX, which increases the diversity within the sequencing run, or the addition of random nucleotides in the used PCRs primers may overcome this issue [48].

### Service vs. in-house methods

Samples processed by a company (see Table 1) undergo a standardized, robust and likely more reproducible workflow compared to a non-specialized laboratory. In addition, setting up a proper library preparation and bioinformatics data analysis pipeline is not trivial and having the analysis performed by experts in the field may help in saving time and effort. However, companies’ services are usually costlier than running the methods in-house if the instruments and personnel are readily available. Also, when thinking of data analysis, it is important to know prior to the experiment, what kind of data and format will be provided by the respective company. Some companies supply both raw sequencing data and even analysed data (e.g. BGI), or only raw data if the analysis is not covered by the service contract; others instead provide only the final analysed output and no raw data (e.g. Adaptive Biotechnologies). Ideally, both formats should be provided, in case one needs or wants to run additional analysis.

An additional option, when possible, may be to use a commercial kit for library preparation and then sequence in-house. This warrants complete oversight and control of sample processing and some companies provide a service of data analysis for self-performed sequencing (e.g. iRepertoire). Per-sample costs may be more expensive than for an in-house established protocol, but likely also less time consuming as the kits are standardized and contain thorough descriptions and advice for troubleshooting. “Open-source” protocols that are used in-house remain the more customizable option and enable for full control of every step of the process.

### Data analysis

Over the past years different tools and strategies have been developed for immune repertoire analysis, of which some have been summarized in previous reviews [14, 15, 56–58]. Other methods, such as IMSEQ [59], TCRklass, iMonitor [60], LymAnalyzer [61] and RTRC [62], have however emerged since. A popular tool is MiXCR (previously MiTCR), developed by Bolotin et al. [33], which allows for a highly customizable analysis of both TCR and immunoglobulin sequences. This is the tool we chose for our analysis, as its parameters may be optimized for different data types, sources and desired outputs. Software specific for analysis of data containing UMIs are MIGEC [50] and pRESTO [63]. The tools listed above are mainly used for primary analysis, as the recovery of TCR sequences from raw data and successive clustering and annotation. LymAnalyzer additionally contains a feature for SNP calling and sequence mutation trees generation for IGs.

Further (secondary) data analysis of the immune repertoire classically involves the calculation of one or more diversity indices [64, 65]. Among the most widely used are the Shannon and the Simpson indices, as well as the Inverse-Simpson and the Gini indices. These differ for example in the consideration they give to factors as the species richness and the evenness of the dataset.

Another typical step of the analysis is the calculation of V and J gene usage in the different samples/datasets. The usage of different V and J genes is indeed not uniform. In literature, there are many examples of biased gene usage [10, 66]. A biased usage of specific genes may also be the result of alterations in the repertoire caused by diseases or other special conditions as organ transplantations.

Different tools for secondary TCR repertoire analysis and diversity estimation are available [67] and a list is available in the repertoire sequencing (Rep-seq) category of the Omic-tools community (https://omictools.com/rep-seq-category) [68]. Recent developments include VDJtools [69], which is capable of analysing outputs from the most common repertoire processing tools described above, and VDJviz [70], a webtool offering similar features as VDJtools. Another tool that provides TCR diversity measures and gene usage statistics computations is the R package “tcR”, which can be used to process the output files format of software as ImmunoSEQ [71, 72], IMSEQ, MiTCR, MiXCR, MIGEC and VDJtools [73]. Other approaches recently developed for estimation of TCR diversity are from Greiff et al., which creates a diversity profile using many diversity coefficients simultaneously [74], from Laydon et al.*,* which introduces a new solution called DivE using rarefaction curves [75] and from Kaplinsky et al., which makes use of a maximum-likelihood based approach without assumptions of the complete repertoire clone richness [76].

When dealing with immune repertoire data, uniformity between samples is important. To this end, especially regarding diversity analyses, down- or re-sampling is a commonly used strategy to generate more comparable data. Similar data types may be easily encountered in the fields of ecology and metagenomics studies. Hence, data analysis packages intended for these disciplines may also be useful for immune repertoire analysis. An example for an interdisciplinary approach already used for TCR data is estimating the total diversity by using the “unseen species model” [77, 78]. The function for this model is provided for example by the “Vegan” R package, together with a series of common diversity measures and estimators [79]. More precise information on low and high complexity data analysis strategies are described in detail elsewhere [80–83].

### Outlook: Single cell methods

Here, we want to briefly cite the main available approaches for single cell analysis of TCRs. There are two options commonly used for the analysis of the TCR repertoire from single cell suspensions: performing overall transcriptome sequencing and extrapolation of TCR information; or using approaches to specifically target the TCR transcript. To our knowledge, the most commonly used commercial workflow for TCR information extraction from transcriptome data is offered through the C1 system machine of the Fluidigm Corporation [84, 85].

Different methods have been established for specific targeting of the TCR transcript, of which some use multiplex primer sets [86]. For example, Han and colleagues used a multiplex PCR approach for TCR α and β chain analysis of FACS sorted cells [8]. Furthermore, they included additional non-interfering multiplex primers in their setup, enabling the parallel study of the expression level of phenotypic traits related to T cells, such as FOXP3, IL17A, TNF and others, thereby providing a more complete picture of the T cells of interest [8]. Another method, named “pairSEQ”, employs an experimental design that divides a sample into different subsets. It then uses combinatorics to evaluate unique TCR αβ chains in every subset [37].

Very recently, Wafergen Biosystems launched a new machine, the ICELL8 single-cell system. This machine may be used with a dedicated kit for TCR sequencing, based on the SMART technology of Takara Bio USA [87]. 10XGenomics, Inc. recently also launched a new dedicated kit for V(D)J analysis. A special note goes to the publicly available instrument and method published by McDaniel and colleagues, which can be used to process millions of cells and to potentially analyse all lymphocyte receptor chains (TCRαβ, TCRγδ, B cell heavy and light chains). This method provides instructions for the construction of a dedicated device, which makes use of an extended concept of the emulsion PCR technology [88], capturing individual molecules using primer-covered beads in droplets within an oil phase, and performing PCR reactions for each bead [89] [90]. This technology, applied to TCR analysis, was previously also published by Turchaninova et al. [91].

For a more detailed summary of single cell approaches to study the immune system, reviews on the topic have been published by Chattopadhyay et al. [92] and Proserpio et al. [93].

## Results

We performed experiments using two of the methods presented above as the most common approaches for bulk immune repertoire sequencing, namely multiplex PCR and 5’RACE-based PCR, using total RNA as starting material. In addition, we compared our results with results provided by the BGI immune repertoire sequencing service, based on a multiplex PCR approach starting from gDNA.

Here, we present some considerations based on our own experiments and analyses, which may help to better understand the methods described until now.

## Replicate correlations

iRepertoire® library preparation and 5’RACE-based PCR were performed in duplicates. Analysis of 5’RACE-based PCR results was performed in parallel for UMI corrected versus non-UMI corrected data. Previous studies have demonstrated the difficulty of detecting the entire TCR diversity of a sample, as it can vary consistently even between very close anatomical locations or between time points. We thus decided to use replicates of the same sample to better assess the stability of TCR diversity, especially when using superficial sequencing [11].

As mentioned before, quantity of starting material and sequencing depth play a major role in defining the extent of the captured TCR diversity. A popular recommendation is to use 100,000 reads to more than efficiently cover the diversity in 10 ng of total RNA [48]. We opted for a superficial sequencing approach (1 million reads for 500 ng total RNA), aiming to detect only the most abundant TCR clonotypes and to determine how many were identified in both duplicates, despite the use of a sequencing depth which likely does not cover the complete diversity present in the sample. For each method (iRepertoire, 5’RACE and 5’RACE UMI corrected) we compared the percentage of clonotypes shared between duplicates (Table 2), assessing their capacity of capturing TCR diversity.

**Table 2:** Percentages of CDR3 nucleotide sequences detected in both duplicates of the same method

Replicates shared clonotypes percentages	α chain			β chain		
	iRepertoire	5’RACE	5’RACE + UMI	iRepertoire	5’RACE	5’RACE + UMI
All clonotypes	36	44	20	35	52	25
Top 300 clonotypes	31	26	27	35	32	37
Top 100 clonotypes	37	31	36	46	51	51
Top 50 clonotypes	45	44	50	38	64	64
Top 20 clonotypes	60	65	70	50	80	75

The percentages are shown for comparisons made between all of the observed clonotypes and between the 300, 100, 50 and 20 most abundant sequences detected by each method. Results include iRepertoire kit data, 5’RACE-based PCR data and data from the same PCR corrected using unique molecular identifiers. Data are shown for both α and β chains

When comparing the total detected repertoire, only 17–52% of observed clonotypes were shared between duplicates (Table 2). However, when we compared only the most abundant clonotypes of each duplicate (the 300, 100, 50 and 20 most abundant clones), we observed a drastic increase in the percentage of shared clonotypes (50–80%). The highest overlap was observed when comparing the most frequent 50 to 20 clonotypes. Thus, despite superficial sequencing depth, we could successfully detect the majority of the most abundant clonotypes in the samples. The increment in the percentage of shared species was even more marked for the UMI corrected data, demonstrating the usefulness of this strategy for error correction. Shared clonotype percentages were comparable between α and β chains.

### Out of frame and stop codon containing sequences

During TCR data analysis it is common to encounter non-functional CDR3 sequences, which are out of frame or stop codon containing sequences. MiXCR labels these sequences with particular symbols, making it possible to exclude these sequences from the clonotype list.

We analysed the percentages of these non-functional sequences in the data obtained from the iRepertoire® kit or 5′ RACE based PCR and in the data provided by BGI, which we re-processed with MiXCR (Table 3). Our results imply that the percentage of both out of frame and stop codon containing sequences is higher in α chain results as compared to β chains, and that out of frame sequences are more commonly detected, as compared to stop codons. iRepertoire results appeared to contain the least non-functional CDR3 sequences. Samples analysed by BGI contained a significantly higher percentage of non-functional CDR3 sequences as compared to other methods, which was to be expected, as gDNA was used as starting material and the sequencing depth was significantly higher. As anticipated, the percentages of non-functional sequences decreased after correcting for UMIs. Percentages were comparable between the two patients we analysed.

**Table 3:** Percentages of detected CDR3 sequences that are out of frame or contain stop codons

Out of frame and stop codons Clonotypes percentages		α chain				β chain			
		IRepertoire	5’RACE	5’RACE + UMI	BGI	IRepertoire	5’RACE	5’RACE + UMI	BGI
Patient 1	Out of frame CDR3%	6.3	9.5	8.4	36.5	1.2	3.1	2.7	16.3
	Stop codons in CDR3%	1.2	2.0	1.5	8.3	0.3	0.8	0.8	4.5
Patient 2	Out of frame CDR3%	7.5	9.0	7.6	39.1	1.0	1.9	1.7	11.3
	Stop codons in CDR3%	1.1	1.5	1.4	7.3	0.2	0.4	0.4	3.2

For each method (we took into account data from iRepertoire kit, 5’RACE PCR, 5’RACE PCR UMI-corrected and data from BGI) we present the percentages of sequences that were considered out of frame or that contain stop codons upon analysis with MiXCR. Data are shown for two patients and for both α and β chain

### Method comparison

Here, we compared results from three different methods performed with the same patient samples, namely BGI service (using gDNA), iRepertoire® kit and the in-house established 5’RACE-based PCR (using RNA). In addition, for 5’RACE data we compared both UMI-corrected and not corrected data. After clonotype grouping and export, all datasets were filtered by retaining only clonotypes detected with two or more counts. To make data sets with different sequencing depths comparable, we decided to assess only the most abundant clonotypes detected by each method. To set a threshold, we determined which methodology provided the lowest total number of detected TCR clonotypes. This applied to UMI-corrected 5′ RACE based PCR (1300–2400 species, depending on chain and patient), which was anticipated due to the strict filtering steps applied during correction. We therefore only considered the most abundant species detected by other methods above this threshold and excluded any sequences below it. Our aim was to determine if high frequency TCR clonotypes and their relative abundancies could consistently be captured using these different library preparation approaches.

**Fig. 3:**
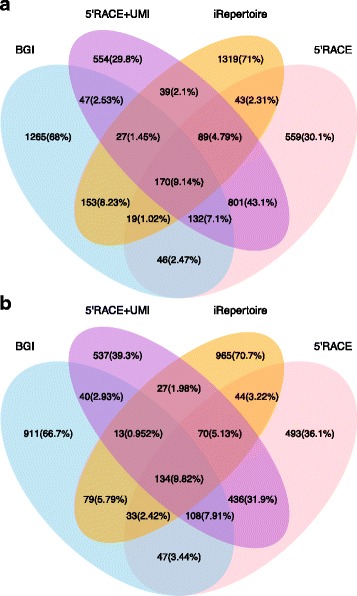
Venn diagram showing the overlap between the most abundant TCR sequences detected by each method. The threshold was defined by the method which detected fewest TCR species (UMI-corrected 5’RACE-based PCR). The diagrams show the number and relative frequency of TCR sequences detected by only one up to all four methods. Sequences that were found only by one method may still be detected by other methods, but may not appear in the most abundant species and are thus not represented here. Data are shown for one patient for both (**a**) α and (**b**) β chains

When analysing the overlap of TCR clonotypes detected by the different methods, we found that less than 10% were captured by all four methods (Fig. 3, Additional file 1: Figure S1). These species were, however, detected at high abundance in all methods. The majority of clonotypes detected by one of the methods were uniquely detected by that particular method (up to 75%), at least among the highly abundant clones. As anticipated, the strongest overlap in species was observed between 5′ RACE-based PCR and UMI-corrected 5′ RACE-based PCR. The percentage of sequences commonly captured by three or all methods increased when considering only the highly abundant clonotypes (Additional file 1: Figure S2). This is in concordance with the observations made for replicate correlations (Table 2). However, clones common to all methods were 19–25% when comparing the top 100 clones and overlap not higher than 37%. These results demonstrate not only the diversity of the TCR repertoire, but also how clonotype abundance within the same sample may vary when using different techniques and correction methods. Indeed, clonotypes detected as highly abundant by only one method, may still be detected in others, but at low counts, resulting in inconsistent information about relative species frequencies. Using strict criteria of UMI correction lead to an involuntary loss of information, reflected by the fact that not UMI corrected clonotypes detected by multiple methods were lost after UMI correction. However, less stringent and conservative methods for UMI correction are available [50] and have recently been discussed by Smith, Heger and Sudbery [94].

### Exemplary analysis of gene usage and diversity

As described in the data analysis paragraph, we assessed the V and J gene usage in our samples for α and β chain.

**Fig. 4:**
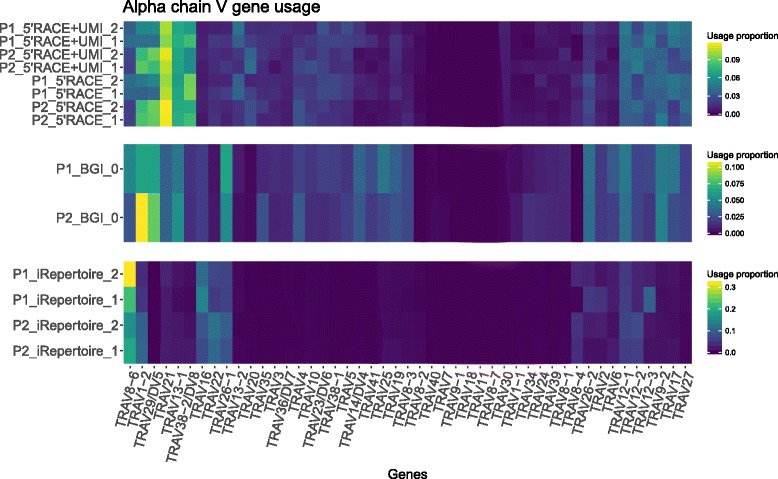
Alpha chain Variable (V) gene usage among methods and replicates. The heat map shows the gene usage proportion inside each sample for the V genes listed at the bottom on the figure. Each of the three parts of the heat map is representative of one of the methods. The samples are described by the patients “P1/2”, the method “iRepertoire/BGI/5’RACE/5’RACE + UMI” and the replicate “1 or 2” or “0” in case of BGI which does not include replicates. The figure was generated using the “ggplot2” R package

The analysis showed a method-dependent gene usage bias for both the V and the J genes of α and β chain (Fig. 4 and additional file 1: Figure S3, respectively). Relative differences in gene usage between the two patients seem to be conserved among different methods, in particular for the most used genes. For example, TRAV8–6 appears to be more used in patient 1 compared to patient 2 and vice versa for TRAV1–2. However, and most importantly, a method-dependent bias in the gene usage was observed. A possible explanation for such differences across methods could be the use of different primers during library preparation. We believe this is a critical observation and an aspect that should be considered prior to attempting to compare results derived from different technologies.

**Fig. 5:**
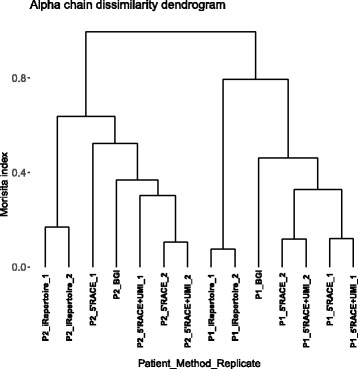
Dissimilarity dendrogram for alpha chain. The distance between patients, methods, and replicates, calculated using the Morisita index, is represented by the dendrograms. The samples are described by the patients “P1/2”, the method “iRepertoire/BGI/5’RACE/5’RACE + UMI” and the replicate “1 or 2”. The figure was generated using the “ggplot2” and the “ggdendro” R packages

In addition to gene usage, we also performed an exemplary analysis to study the diversity among different samples and, in this particular case, methods. Results are shown in Fig. 5.

As expected, samples from the same patient cluster together, as well as replicates from the same method. iRepertoire and 5’RACE PCR results seem to be the most distant from each other, while BGI results appears to cluster more with 5’RACE results, compared to iRepertoire (Fig. 5, Additional file 1: Figure S4).

## Discussion

From our own experience, we learned that different methods are effective in TCR species identification, but to a different extent.

We prefer RNA over gDNA as starting material, because, even if more unstable, RNA contains the final TCR transcript, it does not add noise to the results due to incomplete VDJ-recombination products, and it allows for the use of the 5’RACE method. This is the approach we suggest for library preparation, for two main reasons: (1) it bypasses the amplification biases associated with multiplex PCR and (2) it is suitable for use with UMIs.

In general, we consider low abundance TCR counts as unstable, unless using particularly effective error correction strategies, such as UMIs. These error correction strategies need to be handled with care in order to use the right filtering criteria that fit the needs of the study. If the purposes of the project include analyses of low abundant species, we suggest a strict UMI filtering.

We did not observe a higher overlap with other methods while comparing UMI vs non UMI corrected data. Nevertheless, the concept behind UMI usage remains valid and we believe it should become mandatory in TCR analysis methodologies, in order to avoid PCR distortions, which currently increase the complexity of comparing samples and methods.

To avoid setting up the methods in-house, different kits are commercially available, with or without sequencing service (Table 1). When sequencing in-house, it is important to select the appropriate sequencing depth in relation to the quantity of starting material. More superficial sequencing approaches are cheaper, but may not be able to reflect the entire diversity and are not suitable for rare TCR analysis. However, superficial sequencing may suffice when only abundant clonotypes are of interest. Independent of which method is chosen, one should keep in mind that a uniform quantity and quality of starting material is essential for the positive outcome of any experiment.

Interestingly, we were able to detect some TCR species using low-coverage sequencing, which were not detected within the BGI data. We believe that this may be due to the difficulty of capturing the entire TCR diversity, even with deep sequencing, considering the extremely high variability of the target. Nevertheless, highly abundant species showed a significant overlap between methods. We think that deep sequencing is preferable for studies which aim to extensively analyse the repertoire of cohorts and populations, while low-coverage sequencing might be preferential for studies directed at the identification of already known or abundant clonotypes. Also, due to the high repertoire diversity, performing biological replicates when possible can help reinforce the analysis findings.

## Conclusions

Table 4 summarizes the advantages and disadvantages of every method that we employed in our studies.

**Table 4:** Advantages and disadvantages of the tested techniques

	**+**	**-**
BGI	• Deep sequencing: more complete data • Raw data and analysis provided by the company • Company service: no hands-on work • gDNA as starting material: better clonotype quantification	• Expensive compared to in-house methods • Multiplex PCR amplification bias • Limited PCR and sequencing errors correction • gDNA as starting material: not final TCR product
5’RACE	• In-house method: control of all steps, relatively cheap • No multiplex PCR bias • Unique Molecular Identifiers: correction for PCR and sequencing errors	• Superficial sequencing: less diversity detected • Not high-throughput: small sample number processed per time
iRepertoire®	• Kit: easy and fast hands-on (less than one day) • De-multiplexing and basic data analysis made by the company. FASTA files provided	• Multiplex PCR amplification bias • Limited PCR and sequencing errors correction

It is important to consciously select a method by keeping in mind strengths and weaknesses of each approach, as well as the goal of the scientific project which the method will be applied to. It is also crucial to be careful when comparing relative abundances in data obtained with different methods, as they may be affected by method specific biases as PCR amplification efficiency discrepancies due to different primers.

Many of the mentioned methods may also be applicable to B-cell receptor analyses and as immune repertoire studies are increasing in number and complexity, we believe that an educated choice of methodology is one of the most important steps to achieve the desired results in the growing field of “immunogenetics”.

## Methods

### Sample selection

For benchmarking we used two explanted liver tissue samples from patients with primary sclerosing cholangitis (PSC), which were previously included in a study analysing PSC-related TCR repertoires [95]. PSC is a chronic inflammatory disease of the liver affecting the intra- and extra- hepatic bile ducts. PSC is characterized by high T cell infiltration; thus, it provides a suitable system to study T-cell receptor (TCR) repertoire signatures. To date, it is completely unknown what causes PSC and which is the triggering and/or driving antigen. Detecting disease-associated TCR signatures would be an important step towards the identification of the triggering/driving antigen. Therefore, systematic TCR profiling experiments in the organ of interest are of great importance to further understand the immunogenetics of PSC and of other immune-mediated diseases of unknown etiology.

gDNA and RNA were isolated simultaneously from whole fresh-frozen disease-affected tissue using the AllPrep DNA/RNA Mini Kit from Qiagen.

### iRepertoire ®

As an exemplary method for RNA-based multiplex PCR we selected the iRprofile kit from iRepertoire Inc. We processed both samples in duplicates using 500 ng of total RNA. This kit is highly customizable and offers options for both T and B cell receptor sequencing, different receptor chains (αβ, γδ), gDNA or RNA, mouse or human, and sequencing platforms (Illumina, Roche 454). iRprofile contains separate reactions mixes for α and β chains, each uniquely barcoded. The protocol consists of two consecutive PCRs using multiplex primers specific for the V and J genes of the mentioned chains. The resulting products are then ready for sequencing. The sequencing data may then be sent to the company for demultiplexing. Basic data analysis is also performed and demultiplexed data may be requested as FASTA files.

### 5’RACE

The 5’RACE method we chose is an in-house adaptation of the protocol published by Mamedov et al. 2013 [48]. Briefly, the method entails 5’RACE-based cDNA synthesis using a 5′-template switch adapter containing 12 random nucleotides forming a UMI, followed by two consecutive nested PCRs. During the second PCR, Illumina adaptors are incorporated [96]. Consequently, custom sequencing primers were used in the following MiSeq run. α and β chains were amplified in the same reaction for cDNA synthesis and PCR 1, and they were separated and uniquely barcoded during PCR 2. Samples were processed in duplicates using 500 ng of total RNA.

### BGI

5 μg of gDNA were sent to BGI for both α and β chain immune repertoire analysis (2.5 μg/chain) based on multiplex PCR. BGI performed basic data processing which included data filtering, removal of adapter contamination and low quality reads from raw reads and elimination of sequencing background. Alignment to V/D/J gene segments was carried out separately in IMGT database, and data were realigned for best results. BGI also carried out structural analysis which included CDR sequence and base composition, V/D/J recombination insertion and deletion. Data analysis included immune repertoire profiling and differential analysis of diversity between samples, differential expression analysis of clones between samples and differential expression analysis of clones between groups. In addition to complete data analysis all raw FASTQ files were provided by BGI.

### Sequencing and data analysis

RNA sequencing was performed on an Illumina MiSeq 250PE. The sequencing platform used by BGI was Hiseq2000 100PE.

For data analysis, we used MiXCR (version 2.1.1), obtaining a ranked table of clonotypes including relative species abundances, nucleotide and amino acid CDR3 sequences and respective VDJ alleles as output. Sequences containing the same UMI were grouped under the same UMI signature. For each UMI, only the most abundant sequence was selected, while the others were considered PCR or sequencing errors (script used for UMI filtering is available as Additional file 2). UMI filtered results represent absolute mRNA transcript relative abundances in the original sample. BGI provided data obtained using an older version of the MiXCR software (MiTCR), which is why we reprocessed the raw data with the same version of the software we used for iRepertoire and 5’RACE data. Analysis parameters were optimized based on starting material.

Gene usage analysis was performed using the “geneusage” function of the “tcR” R package, while diversity analysis was performed using the “vegdist” function of the “Vegan” R package.

## Additional files

Additional file 1: Supplementary material. Supplementary figures mentioned in the main manuscript and their titles and legends. **Figure S1.** Venn diagram showing the overlap between the most abundant TCR sequences detected by each method. The threshold was defined by the method which detected fewest TCR species (UMI-corrected 5’RACE-based PCR). The diagrams show the number and relative frequency of TCR sequences detected by only one up to all four methods. Sequences which were found only by one method may still be detected by other methods, but may not appear in the most abundant species and are thus not represented here. Data are shown for two patients and for both α and β chains. **Figure S2.** Venn diagram showing the overlap between the top 300 most abundant TCR sequences detected by each method. For every technique (BGI, 5’RACE PCR, 5’RACE PCR UMI-corrected, iRepertoire kit) the 300 most abundant clonotypes were considered. The diagrams show how many sequences were found to be present also in the most abundant 300 clonotypes of other methods. Sequences which are shown to be found only by one method may still be detected by other methods, but don’t appear in the 300 most abundant species of these. Data are shown for two patients and for both α and β chains. **Figure S3.** Variable (V) and Joining (J) genes usage among methods and replicates. The heat map shows the gene usage proportion inside each sample for the Variable and Joining region alleles listed at the bottom on the figure. Each of the three parts of the heat map is representative of one of the methods. The samples are described by the patients “P1/2”, the method “iRepertoire/BGI/5’RACE/5’RACE + UMI” and the replicate “1 or 2” or “0” in case of BGI which doesn’t have replicates. The figure was generated using the “ggplot2” R package. **a**) Beta chain V genes. **b)** Alpha chain J genes. **c)** Beta chain J genes. **Figure S4.** Dissimilarity dendrogram for beta chain. The distance between patients, methods, and replicates, calculated using the Morisita index, is represented by the dendrograms. The samples are described by the patients “P1/2”, the method “iRepertoire/BGI/5’RACE/5’RACE + UMI” and the replicate “1 or 2”. iRepertoire replicate 2 for beta chain did not satisfy the data analysis quality criteria and was therefore excluded. The figure was generated using the “ggplot2” and the “ggdendro” R packages. (DOCX 3501 kb)

Additional file 2: UMI filtering script (python). The script used to filter the unique molecular identifiers. (PY 4 kb)

## Abbreviations

5’RACE: 5′ rapid amplification of cDNA ends
BCR: B-cell receptor
C: Constant
CDR: Complementary determining region
D: Diversity
dCTP: Deoxycytidine triphosphate
FACS: Fluorescence-activated cell sorting
FOXP3: Forkhead box P3
gDNA: Genomic DNA
HTS: High-throughput sequencing
IL17A: Interleukin-17A
J: Joining
MHC: major histocompatibility complex
Mplex-PCR: Multiplex PCR
mRNA: messenger RNA
NGS: Next generation sequencing
PCR: Polymerase chain reaction
PE: Paired-end
PSC: Primary sclerosing cholangitis
TCR: T-cell receptor
TNF: Tumour necrosis factor
UMI: Unique molecular identifier
V: Variable
