# Supplementary material for: Overview of methodologies for T-cell receptor repertoire analysis
Source: BMC Biotechnol. 2017 Jul 10;17:61. doi: 10.1186/s12896-017-0379-9 (PMC5504616; doi:10.1186/s12896-017-0379-9)
Supplement: Supplementary file 1 — Supplementary material. Supplementary figures mentioned in the main manuscript and their titles and legends. Figure S1. Venn diagram showing the overlap between the most abundant TCR sequences detected by each method. The threshold was defined by the method which detected fewest TCR species (UMI-corrected 5’RACE-based PCR). The diagrams show the number and relative frequency of TCR sequences detected by only one up to all four methods. Sequences which were found only by one method may still be detected by other methods, but may not appear in the most abundant species and are thus not represented here. Data are shown for two patients and for both α and β chains. Figure S2. Venn diagram showing the overlap between the top 300 most abundant TCR sequences detected by each method. For every technique (BGI, 5’RACE PCR, 5’RACE PCR UMI-corrected, iRepertoire kit) the 300 most abundant clonotypes were considered. The diagrams show how many sequences were found to be present also in the most abundant 300 clonotypes of other methods. Sequences which are shown to be found only by one method may still be detected by other methods, but don’t appear in the 300 most abundant species of these. Data are shown for two patients and for both α and β chains. Figure S3. Variable (V) and Joining (J) genes usage among methods and replicates. The heat map shows the gene usage proportion inside each sample for the Variable and Joining region alleles listed at the bottom on the figure. Each of the three parts of the heat map is representative of one of the methods. The samples are described by the patients “P1/2”, the method “iRepertoire/BGI/5’RACE/5’RACE + UMI” and the replicate “1 or 2” or “0” in case of BGI which doesn’t have replicates. The figure was generated using the “ggplot2” R package. a) Beta chain V genes. b) Alpha chain J genes. c) Beta chain J genes. Figure S4. Dissimilarity dendrogram for beta chain. The distance between patients, methods, and replicates, calculated using the Mo [file 12896_2017_379_MOESM1_ESM.docx]

**Overview of methodologies for T cell receptor repertoire analysis**

Rosati E^1^, Dowds CM^1^, Liaskou E^2^, Henriksen EK^3^, Karlsen TH^3,4^, Franke A^1,¶^

**Supplementary material**

α chain

β chain

Patient 1

Patient 2

**Figure S1: Venn diagram showing the overlap between the most abundant TCR sequences detected by each method.**

The threshold was defined by the method which detected fewest TCR species (UMI-corrected 5’RACE-based PCR). The diagrams show the number and relative frequency of TCR sequences detected by only one up to all four methods. Sequences which were found only by one method may still be detected by other methods, but may not appear in the most abundant species and are thus not represented here. Data are shown for two patients and for both α and β chains.

α chain

β chain

Patient 1

Patient 2

**Figure S2: Venn diagram showing the overlap between the top 300 most abundant TCR sequences detected by each method.**

For every technique (BGI, 5’RACE PCR, 5’RACE PCR UMI-corrected, iRepertoire kit) the 300 most abundant clonotypes were considered. The diagrams show how many sequences were found to be present also in the most abundant 300 clonotypes of other methods. Sequences which are shown to be found only by one method may still be detected by other methods, but don’t appear in the 300 most abundant species of these. Data are shown for two patients and for both α and β chains.

b)

a)

**Figure S3: Variable (V) and Joining (J) genes usage among methods and replicates.** The heat map shows the gene usage proportion inside each sample for the Variable and Joining region alleles listed at the bottom on the figure. Each of the three parts of the heat map is representative of one of the methods. The samples are described by the patients “P1/2”, the method “iRepertoire/BGI/5’RACE/5’RACE+UMI” and the replicate “1 or 2” or “0” in case of BGI which doesn’t have replicates. The figure was generated using the “ggplot2” R package. **a**) Beta chain V genes. **b)** Alpha chain J genes. **c)** Beta chain J genes.

c)

**Figure S4: Dissimilarity dendrogram for beta chain.** The distance between patients, methods, and replicates, calculated using the Morisita index, is represented by the dendrograms. The samples are described by the patients “P1/2”, the method “iRepertoire/BGI/5’RACE/5’RACE+UMI” and the replicate “1 or 2”. iRepertoire replicate 2 for beta chain did not satisfy the data analysis quality criteria and was therefore excluded. The figure was generated using the “ggplot2” and the “ggdendro” R packages.
